# Role of Cytosolic Malic Enzyme in Oleaginicity of High-Lipid-Producing Fungal Strain *Mucor circinelloides* WJ11

**DOI:** 10.3390/jof8030265

**Published:** 2022-03-05

**Authors:** Abu Bakr Ahmad Fazili, Aabid Manzoor Shah, Tahira Naz, Shaista Nosheen, Wu Yang, Victoriano Garre, Younis Majeed, Mohammed Khalid Al-Sadoon, Yuanda Song

**Affiliations:** 1 Colin Ratledge Center for Microbial Lipids, School of Agricultural Engineering and Food Science, Shandong University of Technology, Zibo 255000, China; faziliab7@gmail.com (A.B.A.F.); aabidmanzoor87@gmail.com (A.M.S.); nazkhan658@gmail.com (T.N.); shaista_nosheen@yahoo.com (S.N.); neverhangsome@163.com (W.Y.); 2 Departamento de Genética y Microbiología, Facultad de Biología, Universidad de Murcia, 30100 Murcia, Spain; vgarre@um.es; 3 Department of Biotechnology, University of Kashmir, Srinagar 190006, Jammu and Kashmir, India; bhatyounis2@gmail.com; 4 Department of Zoology, College of Science, King Saud University, P.O. Box 2455, Riyadh 11451, Saudi Arabia; msadoon@ksu.edu.sa

**Keywords:** oleaginous fungus, lipid accumulation, *Mucor circinelloides*, malic enzyme, gene overexpression

## Abstract

*Mucor circinelloides*, an oleaginous filamentous fungus, is gaining popularity due to its ability to synthesize significant amounts of lipids containing γ-linolenic acid (GLA) that have important health benefits. Malic enzyme (ME), which serves as the main source of NADPH in some fungi, has been found to regulate lipid accumulation in oleaginous fungi. In the present study, the role of two cytosolic ME genes, *cmalA* and *cmalB,* in the lipid accumulation of the *M. circinelloides* high-lipid-producing strain WJ11, was evaluated. Strains overexpressing *cmalA* and *cmalB* showed a 9.8- and 6.4-fold rise in specific ME activity, respectively, and an elevation of the lipid content by 23.2% and 5.8%, respectively, suggesting that these genes are involved in lipid biosynthesis. Due to increased lipid accumulation, overall GLA content in biomass was observed to be elevated by 11.42% and 16.85% in *cmalA* and *cmalB* overexpressing strains, respectively. Our study gives an important insight into different studies exploring the role of the *cmalA* gene, while we have for the first time investigated the role of the *cmalB* gene in the *M. circinelloides* WJ11 strain.

## 1. Introduction

For decades microbial oil has been regarded as an important substitute for oils derived from plants. Microbial oil was generated commercially for the first time in 1985 but was found to be too expensive compared to the plant source [1]. Later, microbial oil was regarded as an important generator for the production of novel fatty acids that were difficult to be generated via agricultural means [1]. Many oleaginous microorganisms have been reported to produce polyunsaturated fatty acids (PUFAs) that cannot be synthesized by the human body [2,3,4]. Thus, lipogenic fungi are considered vital sources of ω- 6 and ω- 3 PUFAs, which have substantial economic benefit [5,6]. The medicinal significance of PUFAs has been investigated intensively in the past decades [7]. Together, these factors have contributed to the emergence of microbial oil being valuable in terms of industry and economics. Moreover, microbial lipid production is becoming attractive because of its extensive utilization as a biofuel and nutraceutical source [8,9,10,11]. 

In microbial oil-producing organisms like fungi, microalgae, yeast, and bacteria, lipid accumulation takes place under nitrogen depletion and carbon excess conditions [12]. *Mucor circinelloides* is deemed an exemplary oleaginous filamentous fungus for the accumulation of lipids because of the existence of genomic tools, known genomic data, and its capability to generate PUFAs rich in γ-linolenic acid (GLA, 18:3; n-6). A study about recent molecular tools to genetically manipulate various fungi provides important insights about genetic engineering of *M. circinelloides* [13]. 

In *M. circinelloides*, the mechanism of the accumulation of lipids at biochemical and molecular stages has been unfolded by the application of metabolic flux analysis [14], proteomics [15], genomics [16], the activity analysis of key enzymes [17,18], and the influence of metal, phosphate, and calcium ions [19,20]. The pentose phosphate pathway and malic enzyme (ME; EC 1.1.1.40) have been suggested to be chief NADPH suppliers in *M. circinelloides* [17,18]. Employing stoichiometric evaluation, it has been proposed that ME has a vital role in various organisms, even though all the NADPH needed for lipid biosynthesis cannot be provided by it [21]. In the elucidation of the biosynthesis of lipids in lipogenic fungi, the determination of this NADPH source is among one of the major problems of metabolism. Key functions of the NADP^+^-dependent cytosolic malic enzyme have been reported and discussed in various reviews and research articles [1,12,22,23].

Investigation conducted by Zhang et al. [24] showed a 2.5-fold elevation in the accumulation of lipids when the ME gene *malEMc* was overexpressed. On the other hand, when a specific ME inhibitor called sesamol was supplemented in the culture media of *M. circinelloides* CBS 108.16 [25], a reduction from 24% to 2% was noted. Furthermore, when the activity of ME diminishes, lipid accumulation is observed to be throttled in *M. circinelloides* and related fungus-like *Mortierella alpina* [26]. Rodríguez-Frómeta et al. [17] also overexpressed the ME gene *malA* in the CBS 277.49 strain of *M. circinelloides*, but the mutant strain failed to show any elevation in the accumulation of lipids, regardless of the elevation in ME activity and mRNA levels, signifying the presence of other lipid accumulation holdups. Interestingly, in the *M. circinelloides* genome (http://genome.jgi-psf.org/Mucci2/Mucci2.home.html 7 February 2022), using bioinformatic analysis discovery of three putative mitochondrial ME genes (gene ID 78524, 11639 and 166127) and two putative cytosolic genes (gene ID 182779 and 186772), has been performed [27]. 

In the current study, we overexpressed two cytosolic ME genes under the control of a strong promoter in the *M. circinelloides* lipid-overproducing strain WJ11. We named these genes *cmalA* and *cmalB*. Though the *cmalA* gene has been studied in other strains of *M. circinelloides*, we investigated it for the first time in the WJ11 strain. On the other hand, the *cmalB* gene has not been investigated before in any strain of *M. circinelloides,* and to the best of our information, we are investigating it for the first time. We found that overexpression of both genes produced a considerable elevation in ME activity, mRNA levels, and lipid accumulation, although lipid levels increased more significantly in the case of the *cmalA* gene. 

## 2. Materials and Methods

### 2.1. Strains, Growth, and Transformation Conditions

*cmalA* and *cmalB* genes were amplified from the *M. circinelloides* WJ11 strain (CCTCC No. M 2014424). In all the transformation experiments, as a recipient strain, *M. circinelloides* M65, the uracil auxotroph of *M. circinelloides* WJ11, was used. Electroporation was employed to carry out the transformation [28]. YPG or MMC media was used to grow the cultures at 26 °C [29,30]. When needed, 200 mg/mL of uridine was added to the media. For mycelia and colonial growth, pH was regulated to 4.5 and 3, respectively. 

150 mL of Kendrick and Ratledge (K & R) medium [31] was poured into 500 mL baffled flasks, and in it Mc-cmalA-4 (*cmalA* overexpression) and Mc-cmalB-5 (*cmalB* overexpression) strains and wild-type control strain (Mc-cmal-2075) were cultivated independently, for 24 h, with 150 rpm shaking at 28 °C. Cultures from these baffled flasks were then utilized as seed culture to be poured in a 2 L fermenter (BioFlo/CelliGen 115, New Brunswick Scientific, Edison, NJ, USA). The 2 L fermenter contained 1.5 L of altered K & R medium with 80 g glucose/L. Conditions maintained in the fermenter were as follows: shaking = 700 rpm, temperature = 28 °C, pH = 6, and aeration = 0.5 air volume per fermenter volume per minute (*v*/*v* min^−1^). 2 M HCL and 2 M NaOH were utilized for automatic pH maintenance. For the propagation and maintenance of the overexpression plasmids, *Escherichia coli* DH5α cells were used. LB media was used for their growth at 37 °C and 220 rpm shaking [32].

### 2.2. Plasmid Construction

For constructing *cmalA* and *cmalB* overexpression plasmids, two independent pMAT2075 plasmids where used. To enable their chromosomal integration via homologous recombination, they included one kb downstream and upstream *CarRP* sequences flanking *pyrF* genes [33]. From the *M. circinelloides* WJ11 genome, PCR amplicfication of *cmalA* and *cmalB* genes was carried out using primes *cmalA*-1F-*Xho*I/*cmalA*-1R-*Xho*I and *cmalB*-1F-*Xho*I/*cmalB*-1R-*Xho*I, respectively (Supplementary Table S1). Within these primers, the incorporation of 30 bp homologous sequences of XhoI restriction sites was carried out. To generate plasmids pMAT2075-*cmalA* and pMAT2075-*cmalB*, respective PCR fragments were digested with *Xho*I restriction endonucleases and ligated into plasmid pMAT2075 (One-step cloning kit, Takara, Shiga, Japan). These plasmids were then extracted from *E. coli* and PCR analysis was conducted using Ch-1F/Ch-1R and Ch-2F/Ch-2R primers, respectively, for *cmalA* and *cmalB* genes. Moreover, to confirm the sequence of the genes, DNA sequencing was performed. To release overexpression *cmalA* and *cmalB* constructs from their respective plasmids, *Sma*I was used as the digestive enzyme. The released constructs were then transformed into M65 protoplasts, wherein white colonies were produced because of *carRP* locus integration, and where integration of the *carRP* locus did not take place, yellow colonies were generated. The Rodríguez-Frómeta et al. [17] method was used for albino colony selection.

### 2.3. Preparation of Genomic DNA

For the extraction of DNA, K & R medium was used to grow *M. circinelloides* at conditions: shaking = 150 rpm, temperature = 28 °C, and time = 3 days. Under reduced pressure, a Büchner funnel was employed to harvest the mycelium. Distilled water was then used thrice to wash this mycelium. A DNA Quick Plant System kit was employed for the extraction of genomic DNA (Tiangen Biotech Co., Ltd., Beijing, China).

### 2.4. Reverse Transcription-Quantitative PCR (RT-qPCR) to Analyse the Expression of Genes

In a 2 L fermenter Mc-cmalA-4 and Mc-cmalB-5 and control strain Mc-cmal-2075 were cultivated, and subsequently, mycelium was harvested at 6 h, 24 h, 48 h, 72 h, and 96 h to do RT-qPCR analysis. From the collected mycelia, total RNA was extracted by the application of Trizol after grinding mycelia using liquid nitrogen and then transformed into cDNA by using PrimeScriptTM RT reagent kit (Takara) according to instructions of the manufacturer. The quantitative RT-PCR was executed with the help of Light Cycler 96 Instrument (Roche Diagnostics GmbH, Basel, Switzerland) and Maxima SYBR Green qPCR Master Mix (Thermo scientific, Waltham, MA, USA) according to instructions of the manufacturer. Using the method of 2^−ΔΔCt^ and actin as housekeeping gene, data quantification was performed as described previously [34].

### 2.5. Measurement of the Concentration of Nitrogen and Glucose in Culture Medium

The indophenol method was employed to estimate the concentration of ammonium in the culture medium [35]. The glucose oxidase Perid-test kit (Shanghai Rongsheng Biotech Co., Ltd., Shanghai, China) was employed to determine the concentration of glucose as per the guidelines of the manufacturer.

### 2.6. Analysis of CDW and Lipid Accumulation

A Büchner funnel was used to harvest the mycelia that had been cultured for 4 d. The harvested mycelia were subsequently washed with distilled water three times. It was then frozen at −80 °C overnight and a freeze dryer was employed to dry up the mycelia. Then, the cell dry weight (CDW) was calculated gravimetrically [29] and a slightly modified Folch method [36] was employed to perform lipid extraction, which was followed by fatty acid methyl esters (FAME) analysis using gas chromatography (GC). FAME was prepared in tubes using 1 mL of 10% methanol in HCl and kept for 3 h at 60 °C in a water bath. 1 mL of NaCl (saturated) and 2 mL of hexane were utilized to extract methyl esters. After this, a vertical 360 tube rotator was utilized to rotate the tubes for 1 h. This was followed by vortexing and centrifugation at 3000 rpm for 5 min. GC analysis was performed on the top organic hexane layer containing methyl esters. Detectors for flame ionization were incorporated into the gas chromatography machine. A fatty acid standard (Supelco^®^ 37 Component FAME Mix) was used to identify individual chromatographic peaks, and respective chromatographic peaks corresponded to respective fatty acids [37].

### 2.7. Determination of ME Activity

The Hsu and Lardy method [38] was used to determine ME activity with minor modifications. Extraction buffer maintained at pH 7.5, comprising of 50 mM Tris–HCL and 20% glycerol (*w*/*w*) was used during the process.

### 2.8. Statistical Analysis

Three independent experiments were carried out to obtain mean values. Multiple comparison tests using GraphPad Prism (version 7, San Diego, CA, USA) preceded by one-way/two-way ANOVA (wherever applicable) was employed to perform statistical analysis. *p* < 0.05 was considered significantly different.

## 3. Results

### 3.1. Genetic Engineering for Generation of cmalA and cmalB Overexpressing M. circinelloides Strains

The *cmalA* (scaffold00036.12) and *cmalB* (scaffold00049.37) genes were retrieved from *M. circinelloides* WJ11 genomic data. Using plasmid pMAT2075 (carrying the strong promoter zrt1 and *pyrF* gene as a selectable marker) [28], the overexpression recombinant strains of *cmalA* (pMAT2075-*cmalA*) and *cmalB* (pMAT2075-*cmalB*) genes were generated to assess their role in lipid production. The M65 strain (*M. circinelloides* WJ11 uracil auxotroph strain) was used as the recipient strain to transform pMAT2075-*cmalA* and pMAT2075-*cmalB*. Uridine auxotrophy in the M65 strain was complemented by the *pyrF* gene present in the plasmids. Mc-cmalA-4 and Mc-cmalB-5 were chosen as independent transformants, respectively, for the *cmalA* and *cmalB* genes. The Mc-cmal-2075 strain, having wild-type features, was used as the control strain.

**Figure 1:**
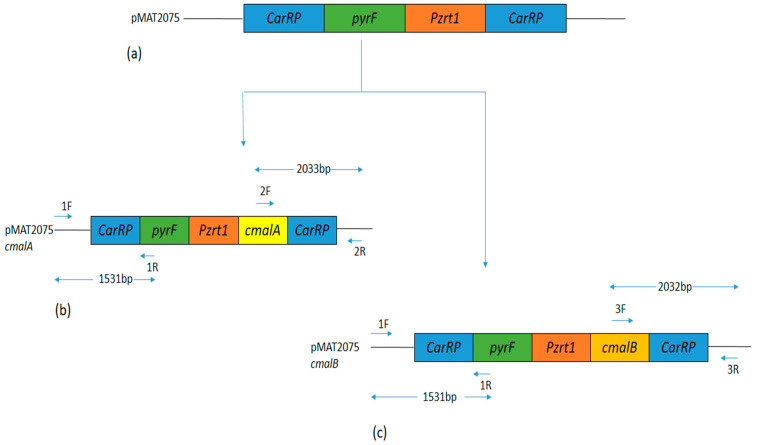
Overexpression of *cmalA* and *cmalB* genes. (**a**) Plasmid structure of plasmid pMAT2075. (**b**) Plasmid structure of pMAT2075-*cmalA* in Mc-cmalA-4 strain. Positions for primers 1F, 1R, 2F, and 2R are indicated. (**c**) Plasmid structure of pMAT2075-*cmalB* in Mc-cmalB-5 strain. Positions for primers 1F, 1R, 3F, and 3R are indicated.

**Figure 2:**
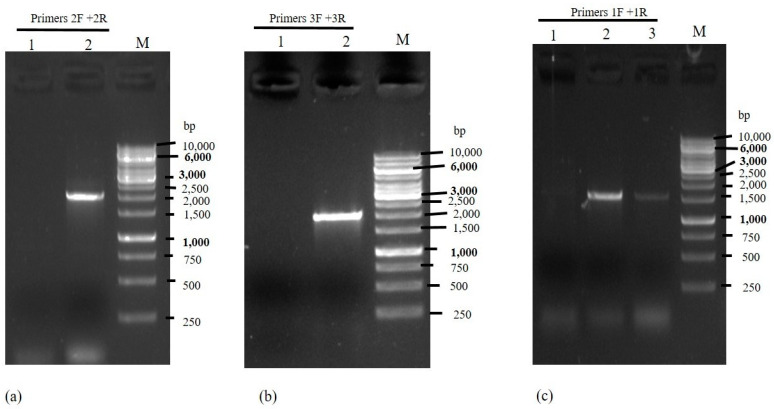
PCR amplification in Mc-cmal-2075, Mc-cmalA-4, and Mc-cmalB-5 strains. (**a**) PCR amplification of *cmalA* gene and 3′ *carRP* region using primer pair 2F/2R in overexpressing transformant Mc-cmalA-4 strain. Lane M: Marker. Lane 1 represents the control, Lane 2 shows the result of the PCR amplification that confirmed the presence of *cmalA* gene in the overexpressing transformant. (**b**) PCR amplification of *cmalB* gene and 3′ *carRP* region using primer pair 3F/3R in overexpressing transformant Mc-cmalB-5 strain. Lane M: Marker, Lane 1 represents the control, Lane 2 shows the result of the PCR amplification that confirmed the presence of *cmalB* gene in the overexpressing transformant. (**c**) PCR amplification of *pyrF* genes and 5′ carRP regions in Mc-cmal-2075, Mc-cmalA-4, and Mc-cmalB-5 strains using primer pairs 1F/1R. Lane M: Marker. Lane 1 represents the control. Lanes 2 and 3 show presence of *pyrF* gene in Mc-cmalA-4 and Mc-cmalB-5 strains, respectively.

Verification of the integration of transforming fragments into the *M. circinelloides* genome was conducted with the help of PCR. The amplification of the 2033 bp band with the primer pair 2F and 2R indicated the integration of the *cmalA* gene in the *carRP* locus of M65 (Figure 1b and Figure 2a). Similarly, the amplification of the 2032 bp band with the primer pair 3F and 3R indicated the integration of the *cmalB* gene in the *carRP* locus of M65 (Figure 1c and Figure 2b). Moreover, the integration of the *pyrF* gene into the *M. circinelloides* genome in Mc-cmalA-4 and Mc-cmalB-5 strains was confirmed by PCR amplification with the primer pairs 1F and 1R producing a band size of 1531 bp (Figure 1b,c and Figure 2c). Altogether, these outcomes authenticated the incorporation of the *cmalA* and *cmalB* genes into the *carRP* locus of the *M. circinelloides* genome under the regulation of the strong promoter *pzrt1* in the Mc-cmalA-4 and Mc-cmalB-5 recombinant strains.

### 3.2. The Expression Levels of cmalA and cmalB Genes in Overexpressing Strains

**Figure 3:**
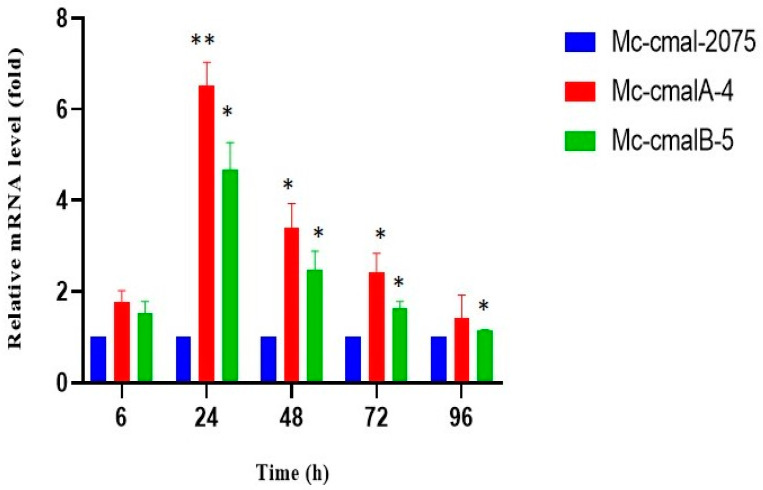
Levels of expression of *cmalA* and *cmalB* genes in control strain Mc-cmal-2075 and overexpressing strains Mc-cmalA-4 and Mc-cmalB-5. Values are the mean of three biological replicates. Error bars represent the standard error of the mean. Asterisks indicate significant differences: * *p* < 0.05 and ** *p* < 0.01.

To evaluate and compare the mRNA levels of the *cmalA* and *cmalB* genes in the respective Mc-cmalA-4 and Mc-cmalB-5 strains and the control strain Mc-cmal-2075, reverse transcription quantitative PCR (RT-qPCR) was performed. It was carried out at 6, 24, 48, 72, and 96 h of growth using primer pairs *cmalA-3*-F/*cmalA-3*-R and *cmalB-3*-F/*cmalB-3*-R for the *cmalA* and *cmalB* genes, respectively (Supplementary File Table S1). In comparison to the control strain, mRNA levels of the *cmalA* and *cmalB* genes in Mc-cmalA-4 and Mc-cmalB-5 overexpression strains were found to be considerably elevated. The level of expression at 24 h was observed to be 6.58- and 4.78-fold for Mc-cmalA-4 and Mc-cmalB-5 strains. A decrease in the trend was noted after 24 h, but elevation in comparison to the control strain was maintained throughout the process of fermentation, validating the overexpression of respective genes in the recombinant strains (Figure 3). During expression analysis across various specified hours, MC-cmal-2075 was used as reference and its relative expression value was taken as 1. 

### 3.3. ME Activity Analysis of cmalA and cmalB Overexpressing Strains

**Figure 4:**
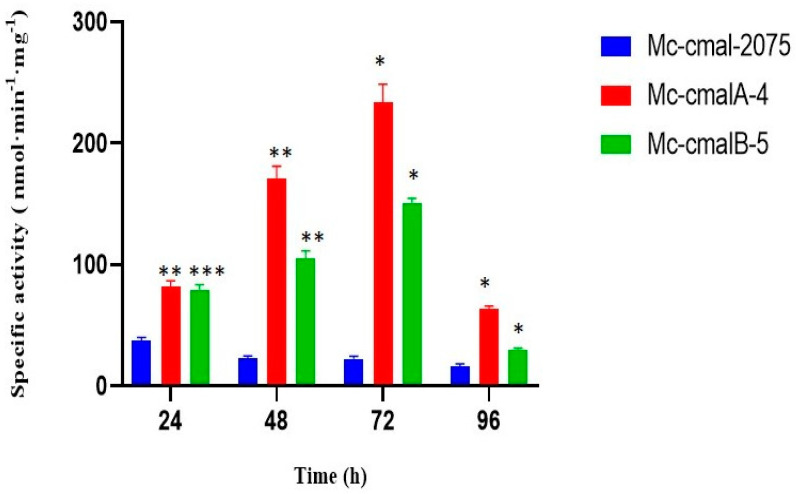
Specific ME activity of the control strain Mc-cmal-2075 and overexpressing strains Mc-cmalA-4 and Mc-cmalB-5. Values are the mean of three biological replicates. Error bars represent the standard error of the mean. Asterisks indicate significant differences: * *p* < 0.05, ** *p* < 0.01, *** *p* < 0.001.

The trend of specific ME activity in overexpressing strains (Mc-cmalA-4 and Mc-cmalB-5) compared to the control strain (Mc-cmal-1557) at 24, 48, 72, and 96 h is shown in Figure 4. Both overexpressing strains showed higher specific ME activity than the control strain throughout the whole culture. In addition, a consistent increase in specific ME activity for both overexpressing strains was observed up to 72 h of culture, whereas the control strain showed a reduction in this activity as the culture progressed. At 72 h of culture, the Mc-cmalA-4 and Mc-cmalB-5 strains showed a 9.8- and 6.4-fold elevation in specific ME activity, respectively, compared to the control strain. Despite the fact that a dip in ME activity in overexpressing strains was observed at 96 h, it was noted to be greater than in the control strain.

### 3.4. Cell Growth and Lipid Accumulation in cmalA and cmalB Overexpressing Strains

**Figure 5:**
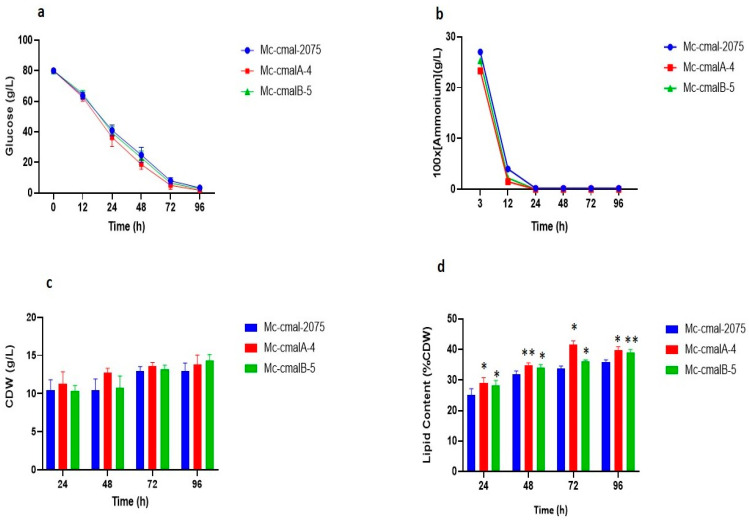
Determination of cell growth of lipid content analysis of recombinant strains (Mc-cmalA-4 and Mc-cmalB-5) and control strain (Mc-cmal-2075) at specific time points. (**a**) Concentration of glucose, (**b**) concentration of ammonium, (**c**) cell dry weight (CDW), (**d**) lipid content. Values are the mean of three biological replicates. Error bars represent the standard error of the mean. Asterisks indicate significant differences: * *p* < 0.05 and ** *p* < 0.01.

The effect of *cmalA* and *cmalB* overexpression on cell dry weight (CDW), lipid accumulation, and ammonium and glucose concentrations were analysed (Figure 5) in 96-h cultures. CDW increased with the incubation, and it was found to be maximum at 96 h for the overexpressing and control strains (Figure 5c). The concentration of glucose was found to be sufficient throughout the fermentation process, while the rate of consumption of glucose was found to be slightly faster in overexpression strains compared to the control strain (Figure 5a). However, nitrogen was depleted from the medium between 12 and 24 h, while lipid accumulation increased (Figure 5b). In the Mc-cmalA-4 and Mc-cmalB-5 strain, compared to the control strain Mc-cmal-2075, at 72 h, lipid accumulation was increased by 23.2% (from 34% in the control strain to 42% in the overexpressing strain) and 5.88% (from 34% in the control strain to 36% in the overexpressing strain), respectively (Figure 5d).

The fatty acid profiles of the recombinant and control strains are shown in Table 1. At 96 h, the GLA content in total fatty acids of Mc-cmal- 2075, Mc-cmalA-4, and Mc-cmalB-5 strains was 9.91%, 9.88%, and 10.35%, respectively. Since overexpression strains generated more lipid content than the control strain, the overall GLA content in the biomass of overexpression strains was noted to be higher than in the control strain. GLA content in biomass was noted to be elevated by 11.42% (from 3.5% to 3.9%) and 16.85% (from 3.5% to 4.09%) in Mc-cmalA-4 and Mc-cmalB-5 strains, respectively.

## 4. Discussion

The conditions of the culture and levels of nutrients are deemed to be important influencers for lipid accumulation. Commercially proficient lipid production necessitates the development of recombinant strains that can accumulate elevated amounts of lipids, independent of the environmental fluctuations. So far, several genetic engineering techniques have been employed to boost lipid production via overexpression or the deletion of important genes and transporters such as malate and citrate transporters [39,40,41,42,43], AMP-activated protein kinase [44,45], the pentose phosphate pathway [18,46,47], Acyl CoA: Diacylglycerol Acyltransferase [48], lipases [49,50,51], malic enzymes [17,24], and the utilization of lignocellulosic biomasses [52].

When ME genes were overexpressed in *M. circinelloides*, lipid accumulation was found to be enhanced 2.5-fold [24], while Rodríguez-Frómeta et al. [17] found no increase in the accumulation of lipids. The difference between these two studies was that the former utilized a self-replicative plasmid resulting in the generation of an unstable and industrially infeasible strain, whereas the latter generated *M. circinelloides* strains that were genetically stable because of the incorporation of the ME gene in the genome’s precise locus. Rodríguez-Frómeta et al. [17] developed an important scheme for the replacement of the gene, preventing the biosynthesis of carotenoids so that their strain could be utilized for the production of a biomass suitable for biodiesel transformation. This replacement strategy generated transformants of two types; yellow transformants in which the gene did not integrate at the desired location and white transformants lacking carotene with gene integration at the desired location. This approach, therefore, added an important tool for *M. circinelloides* research and for probable applications in biotechnology. In our study, we have also utilized the method of Rodríguez-Frómeta et al. [17]. To give an insight into different results produced during the works of Zhang et al. [24] and Rodríguez-Frómeta et al. [17], Hao et al. [53] overexpressed the ME gene *malE1* in a related oleaginous fungus *Mortierella alpina*, and found that the content of fatty acid was elevated by 30%. Their results suggested that in oleaginous fungi, ME has an important role in the biosynthesis of fatty acids but is not the only rate-limiting enzyme. The ME gene (GenBank: DQ975377.1) from *M. circinelloides* has also been incorporated and overexpressed in *Rhodotorula glutinis*, an oleaginous yeast, and it has resulted in the elevation of lipid accumulation by 18.74% [54]. Other studies have shown that ME is not the major enzyme responsible for NADPH generation, but it does have a role in the synthesis of fatty acids [14,55]. In the current study, we have demonstrated that lipid accumulation increased when *cmalA* and *cmalB* genes were put under the regulation of a strong promoter zrt1. In the case of the *cmalA* gene, lipid accumulation was elevated by 23.2%, while for *cmalB* there was an elevation of 5.8%. By observing the trend of ME activity at 96 h, it becomes clear that some factors limit its persistent activity. It is hypothesized that the ME-cleaving enzyme may be responsible for the loss of activity [24].

The overexpression of *cmalA* and *cmalB* genes elevated the lipid content of the cell dry weight by 23.2% (from 34% to 42%) and 5.88% (from 34% to 36%), respectively, even though the activity of ME was elevated by 9.8- and 6.4-fold in respective genes. These outcomes indicate that the enhanced activity of ME did not necessarily result in an adequate elevation in NADPH levels required for the synthesis of fatty acids. Alternately, other pathways of metabolism may provide NADPH for lipid production. Thus, it is probable that the role of ME in the synthesis of fatty acids, perhaps, would not be as vital as suggested earlier [1,24]. Several researchers have exhibited that, among some yeasts or molds, ME overexpression considerably boosts the accumulation of lipids [24,53,54]. The ME recombinant strain of bacteria *Rhodococcus josti*, has been found to enhance lipid accumulation [56]. In microalgae, the mitochondrial malic enzyme of *P. tricornutum*, was found to significantly influence the accumulation of lipids in *P. tricornutum* and *C. pyrenoidosa* [57,58,59]. *C. protothecoides* and *Nannochloropsis salina* has also shown elevation in lipid synthesis when their malic enzymes were overexpressed [60].

Among plant cells, there is a lot of ambiguity about the effect of ME activity on accumulation of lipids. In the case of plants, ME is considered an ubiquitous enzyme having a role in various metabolic pathways such as photosynthesis, stress responses, and the development and growth of plants [61,62,63]; although, various roles of ME in plants still remain unidentified [64]. Hence, it may be difficult to find the gene or genes encoding for ME, having a role in NADPH generation aimed at the biosynthesis of fatty acids. However, a study on the development of embryos in rapeseed has shown that ME may have a role in the synthesis of fatty acids [65].

## 5. Conclusions

This is the first study to investigate the role played in the accumulation of lipids by cytosolic ME genes *cmalA* and *cmalB* in the *M. circinelloides* WJ11 strain, though previously the *cmalA* gene has been evaluated for its role in the accumulation of lipids in the CBS 277.49 strain. The study of cytosolic ME genes in the WJ11 strain revealed that the *cmalA* gene has a more significant role in the accumulation of lipids compared to the *cmalB* gene. In the case of both the genes, the activity of ME is found to be significantly reduced in the end. In conclusion, it can be said that, like some highly competent oleaginous yeasts which retain the activity of ME for a long time, *M. circinelloides,* if engineered to repel ME degradation, can also accumulate a high amount of lipid (~70%). If our study is analyzed within the prism of previous studies, our results further emphasize that ME is not the only rate-limiting enzyme, though it has a significant role in the synthesis of fatty acids.

## Figures and Tables

**Table 1:** Composition of fatty acids (%, *w*/*w* of total fatty acids) * in Mc-cmal- 2075, Mc-cmalA-4, and Mc-cmalB-5 strains.

Strains	Time (h)	16:0	16:1	18:0	18:1	18:2 (LA)	18:3 (GLA)
Mc-cmal-2075	24	17.44 ± 0.3	2.61 ± 0.2	4.90 ± 0.1	39.10 ± 0.9	11.01 ± 0.8	13.59 ± 0.4
	48	20.18 ± 0.5	2.92 ± 0.2	5.18 ± 0.3	41.12 ± 1.0	10.74 ± 0.6	10.80 ± 0.2
	72	22.55 ± 0.7	3.41 ± 0.1	4.62 ± 0.3	41.88 ± 2.1	11.09 ± 0.6	9.76 ± 0.3
	96	22.59 ± 0.5	4.00 ± 0.3	3.93 ± 0.1	42.94 ± 1.5	12.41 ± 0.3	9.91 ± 05
Mc-cmalA-4	24	17.96 ± 0.4 *	1.92 ± 0.2 *	4.89 ± 0.9	35.37 ± 0.8 *	11.48 ± 0.5 **	12.72 ± 0.7 *
	48	21.72 ± 0.4 **	2.57 ± 0.1 *	5.01 ± 0.6 *	40.38 ± 1.5 **	10.45 ± 0.3 *	10.68 ± 0.2 **
	72	21.00 ± 1.1 *	2.79 ± 0.2 **	4.46 ± 0.3	41.00 ± 1.5 **	10.56 ± 0.2	9.05 ± 0.6 *
	96	22.95 ± 0.7 **	3.59 ± 0.1 *	4.18 ± 0.2 *	41.97 ± 1.3 *	11.36 ± 0.3	9.88 ± 0.6 *
Mc-cmalB-5	24	18.95 ± 0.7 *	2.14 ± 0.3 *	4.88 ± 0.4	39.20 ± 0.7 *	12.92 ± 0.2 *	13.49 ± 0.4 *
	48	21.82 ± 0.6 **	2.99 ± 0.2 *	6.95 ± 0.4 *	43.74 ± 1.3 *	11.89 ± 0.3 **	10.89 ± 0.3 **
	72	22.75 ± 0.9 **	3.23 ± 0.4	5.31 ± 0.3	45.06 ± 1.5 **	11.45 ± 0.4 **	10.30 ± 0.2 *
	96	23.66 ± 0.2 ***	4.89 ± 0.3 *	4.94 ± 0.2 *	45.10 ± 1.8 *	12.26 ± 0.1	10.35 ± 0.4 *

* The fatty acid composition displayed at different time point. The values are means ± standard deviations of three independent experiments. Asterisks indicate significant differences: * *p* < 0.05, ** *p* < 0.01; *** *p* < 0.001.

## Data Availability

All relevant data generated or analyzed during this study are included in this article.

## Supplementary Materials

The following supporting information can be downloaded at: https://www.mdpi.com/article/10.3390/jof8030265/s1, Table S1. Sequences of primers utilized in this study.
