# Supplementary material for: Role of Cytosolic Malic Enzyme in Oleaginicity of High-Lipid-Producing Fungal Strain Mucor circinelloides WJ11
Source: J Fungi (Basel). 2022 Mar 5;8(3):265. doi: 10.3390/jof8030265 (PMC8955760; doi:10.3390/jof8030265)
Supplement: Supplementary file 1 [file jof-08-00265-s001.zip › jof-1611032-supplementary.pdf]

Table S1. Sequences of primers utilized in this study.

Primer name	Primer sequence (5'-3')
cmalA-1F-*Xho*I	CAAAATAACTAAATTACGTAGCTAGCCTCGAGATGGATCCTGCTAAACGTCAAAGAC
cmalA-1R-*Xho*I	CTCATCTTTCCCTGTCTGCCTCGAGCTACAATTTACCAGCTTGCTG
cmalB-1F-*Xho*I	CAAAATAACTAAATtACGTAGCTAGCCTCGAGATGGCTCCTTCATCCTACATTGC
cmalB-1R-*Xho*I	CTCATCTTTCCCTGTCTGCCTCGAGCTAAATAGAGGATGGCAAGGATCCG
1F	GATAAGCATAAACCAGATCTG C
1R	GAGATCTCGACGTATTCAGCG
2F	GAAAGAGAATCAAGTGTCTCC
2R	GTATCTGACATAGTCGAGCTTG
3F	ATACTTCCAAGCTGAGTTTG
3R	GTATCTGACATAGTCGAGCTTG
*cmalA*qPCR-F	TCAAAAAGCTCGCGCCCTCA
*cmalA*qPCR-R	AGGGGCAGCCAAGAAGGGAT
*cmalB*qPCR-F	TCAACCGTCTTGCCGCCATT
*cmalB*qPCR-R	TAGGCTTAGCCAACACGCCG
Actin-F	GATGAAGCCCAATCCAAGAGAGGT
Actin-R	TCTTCTCCGGTTGGACTTGGG

* Restriction enzyme sites (6 bp nucleotides) are underlined.
